# Supplementary figures and images for: Sources and sinks of influenza A virus genomic diversity in swine from 2009 to 2022 in the United States
Source: J Virol. 2025 Aug 26;99(9):e00541-25. doi: 10.1128/jvi.00541-25 (PMC12455956; doi:10.1128/jvi.00541-25)

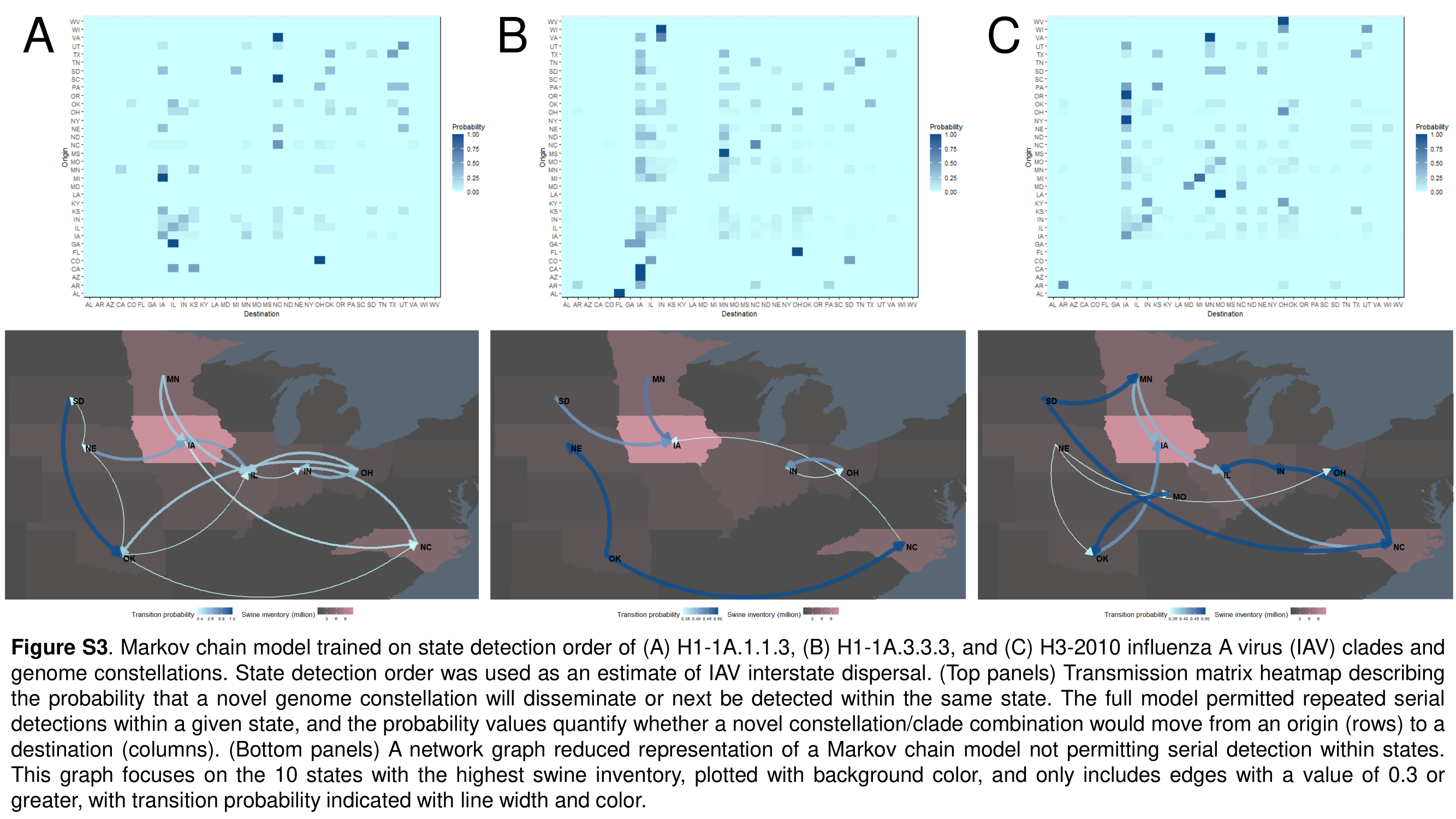

Supplement: Fig. S3 — Markov chain model trained on state detection order of H1-1A.1.1.3, H1-1A.3.3.3, and H3-2010 influenza A virus (IAV) clades and genome constellations. [file jvi.00541-25-s0003.tiff]
